# Estimating Lean Body Weight in Adults With the PAWPER XL-MAC Tape Using Actual Measured Weight as an Input Variable

**DOI:** 10.7759/cureus.29278

**Published:** 2022-09-17

**Authors:** Mike Wells, Lara N Goldstein

**Affiliations:** 1 Department of Emergency Medicine, Florida Atlantic University Charles E. Schmidt College of Medicine, Boca Raton, USA; 2 Department of Emergency Medicine, HCA Florida Aventura Hospital, Aventura, USA

**Keywords:** drug dosing, obesity, pawper tape, pharmacology, lean body weight

## Abstract

Introduction

Accurate drug dose calculation in obese patients requires an estimation of lean body weight (LBW) for dosing hydrophilic medications. Inaccurate weight estimates during the management of critically ill obese adults may contribute to inaccurate drug doses and consequential poor outcomes. Existing methods of LBW estimation or measurement may be very difficult or impossible to use during emergency care. A point-of-care model that could provide rapid, accurate estimates of LBW would, therefore, be of significant clinical value.

Methods

A model was derived based using the adult version of the PAWPER XL-MAC tape. This derived model used recumbent length and measured total body weight (TBW) to estimate LBW. The derived model was used to generate LBW estimations in a random sample from National Health and Nutrition Examination Survey (NHANES) datasets (n=33,215). The benchmark outcome measure was to achieve >95% of LBW estimations within 20% of DXA-measured fat-free mass (P20>95%) and >70% of estimations within 10% of DXA-measured fat-free mass (P10>70%).

Results

The new model achieved a P20 of 99.7% and a P10 of 86.4% for LBW in the pooled sample and exceeded the minimum accuracy standards. This accuracy was maintained in both sexes, all ages, all ethnic groups, all lengths and in all habitus types.

Conclusions

The modified PAWPER XL-MAC model, using TBW as an input variable, proved to be an accurate method of LBW estimation. It could potentially have an important role in facilitating emergency drug dose calculations in critically ill or injured obese adult patients.

## Introduction

Background

Weight-based drug therapy in obese patients is complicated by the need to use the appropriate weight scalar for each drug administered [1,2]. In general, lipophilic drugs should be dosed to total body weight (TBW) and hydrophilic drugs to ideal body weight (IBW) or lean body weight (LBW) [3,4]. Unfortunately, LBW cannot be measured in critically ill patients and can be difficult to estimate during a time-sensitive emergency and critical care [5]. The most common methods of estimating LBW, such as the Boer formula and the Janmahasatian formula, require complex mathematical calculations which can be time-consuming and prone to error, especially during emergencies [6,7]. Recent studies have looked at adapting paediatric weight estimation systems, in particular the PAWPER XL-MAC tape, to estimate TBW and LBW in adults [8-11]. The PAWPER XL-MAC is a tape that is used to measure the recumbent length and mid-arm circumference (MAC), which are used to determine the weight estimates. The weights are read directly off the tape, with no need for calculations. The initial results have been promising but have shown a lower accuracy in morbidly obese patients than in other patients. The reduction in the accuracy of LBW estimations by the PAWPER XL-MAC tape in this subgroup of patients is primarily because of reductions in the accuracy of the TBW estimations (TBW is an important determinant of LBW in all body types) [12]. Therefore, if a more accurate estimation of TBW, or an actual measured TBW, was available, then it might be possible to increase the accuracy of LBW estimations in morbidly obese patients. The advantage of adapting a method such as the PAWPER XL-MAC tape for this purpose is that it requires no calculations for the estimation which can be performed in a few seconds [13].

Importance

The ability to estimate LBW easily and accurately is both relevant and important. It is relevant because obesity is common, with a prevalence of more than 37% in the USA, and doctors providing emergency or critical care will frequently encounter obese patients [14,15]. The ability to estimate LBW is important because the use of TBW instead of LBW as a scalar for hydrophilic drugs can result in substantial overdose with concomitant potential for patient harm [15,16]. Weight estimates that deviate from the reference standard by as little as 10% can be associated with medication error and potential patient harm [17]. Therefore, accurate estimations of TBW and LBW are required to minimise the potential for patient harm.

In addition, improved access to methods of rapidly estimating LBW at the patient’s bedside could increase the use of correct dose scaling in clinical medicine as well as drug-related research. This could lead to a reduction in medication errors in obese patients and enhanced patient safety [15].

Goals of this study

The aim of this study was to evaluate the accuracy of LBW estimations using the PAWPER XL-MAC tape model in a new way, when actual TBW, rather than an estimate of TBW, is included in the model estimation methodology.

## Materials and methods

This was a “virtual study,” in which the estimation of LBW by a new method of using the PAWPER XL-MAC method, was evaluated using data from a large database.

Patients

The National Health and Nutrition Examination Survey (NHANES) datasets from 1999-2000 to the 2017-2018 surveys were downloaded from the Centers for Disease Control (CDC) website [18]. The downloaded data included the following variables: sex, race, age, total body weight (TBW), fat-free mass (FFM) measured using dual-energy x-ray absorptiometry (DXA), height, and body mass index (BMI). Data from participants under the age of 16 years were excluded. Individuals with incomplete or missing data were also excluded from the analysis. The datasets were pooled for analysis.

Ethics

The National Health and Nutrition Survey Examination studies were each approved by the Research Ethics Review Board of the National Center for Health Statistics. This current study was exempted from additional IRB review as the study only utilized anonymized, publicly available data.

LBW estimation model

**Figure 1:**
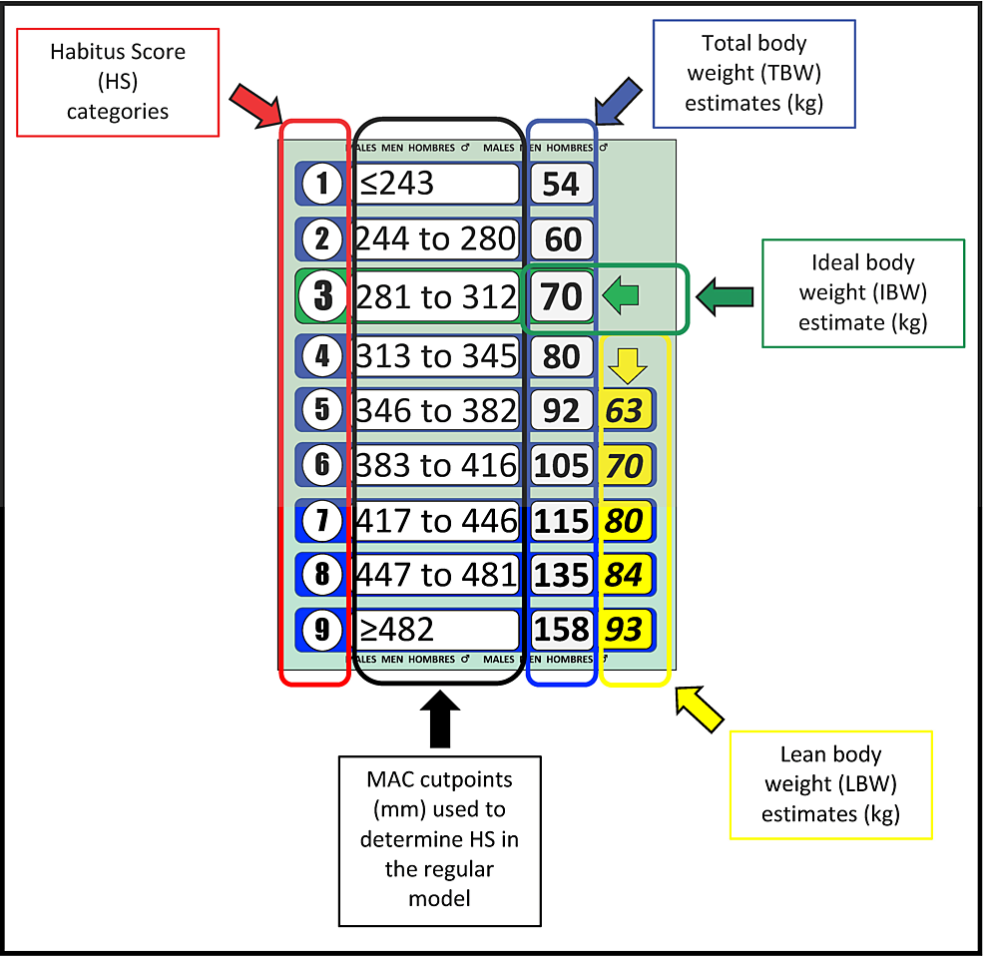
Details of the data contents of each length segment of the adult version of the PAWPER XL-MAC tape. The total body weight (TBW), ideal body weight (IBW) and lean body weight (LBW) estimates are shown for each habitus score (HS) category. LBW estimates are only shown for obese adults (HS 5 and above) as this is the subpopulation in which LBW would be needed for dose calculations for hydrophilic drugs.

The PAWPER XL-MAC tape is a 196 cm long tape, designed to be able to predict TBW, IBW and LBW in male and female adolescent and adult patients. There is insufficient reference data to establish weight estimation parameters in patients taller than 196cm. The tape is divided into 3 cm length segments. Figure 1 shows an example of one of the segments of the adult version of the PAWPER XL-MAC tape. Each segment provides weight estimation information relevant to patients of that length. In the original model, the patient’s length is used to determine the appropriate length segment to use for that patient. This is accomplished by laying the tape alongside the patient, aligning the top end with their vertex, and observing where their heel crosses the tape. The tape is then used to measure their MAC, which determines their habitus score (HS). The weight estimation information can then be read directly from the tape in the patient's length segment, at the appropriate HS.

**Figure 2:**
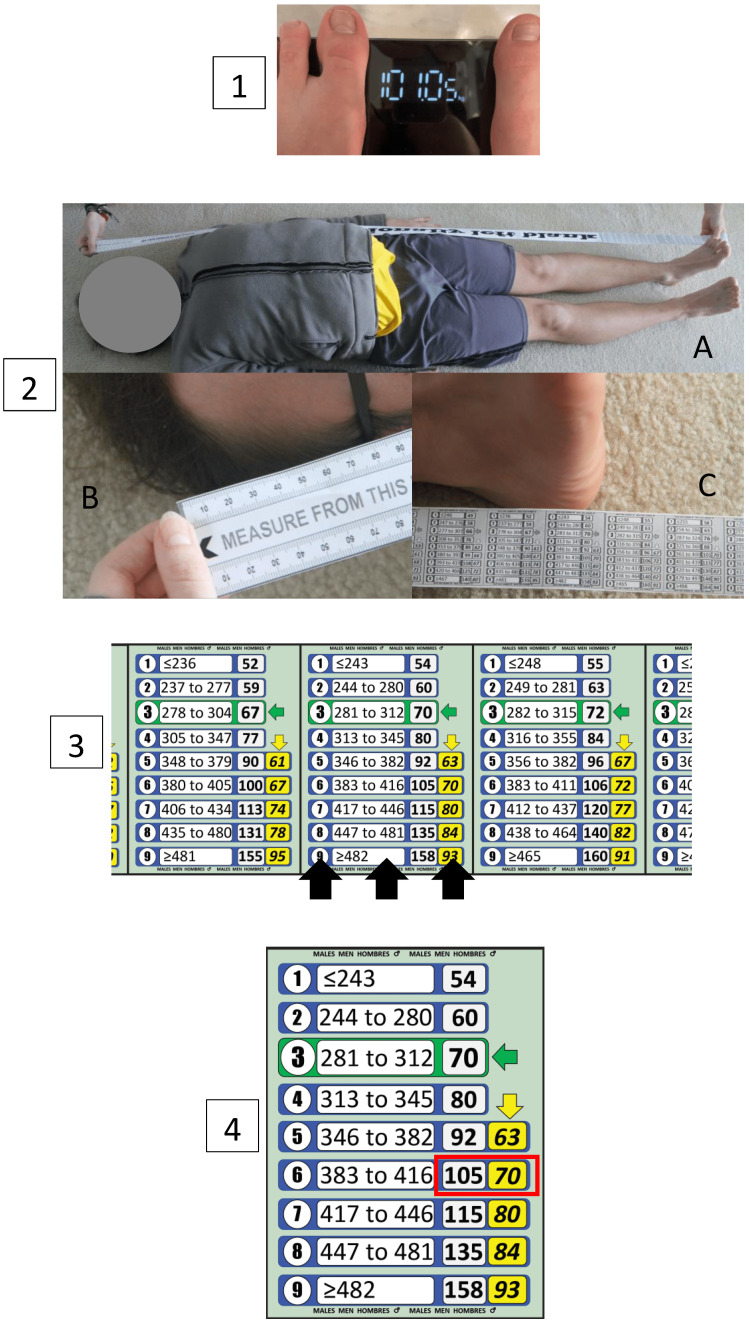
How the adult version of the PAWPER XL-MAC tape is used in this current model to estimate lean body weight. Panel 1 – if a measured weight is known, or if a patient can be weighed then this weight (in kg) should be used in the process to estimate lean body weight (LBW). Panel 2 – the first step is to measure the patient’s length with the tape, from vertex to heel, to determine which length segment must be used for the subsequent steps. (A) Tape positioning (two users in this case). (B) Alignment of the tape at the vertex. (C) Tape alignment at the heel. Panel 3 – the length segment at the heel should be noted for the final step (indicated by the black arrows). Panel 4 – the total body weight estimate closest to the patient’s actual weight should be used to determine their habitus score (in this example, the total body weight of 105kg in the HS 6 category is closest to the patient’s weight of 101kg - indicated by the red rectangle). The lean body weight estimate in that habitus score category would then apply to this patient (an estimated lean body weight of 70kg).

The model described and evaluated in this paper varies slightly from the original methodology. Since estimations of LBW are likely to be more accurate if actual rather than estimated TBW is used in the estimation process, a modified model to estimate LBW was developed. In this new model, the tape is used to measure the patient’s length to identify their length segment, in the same way as the original method. However, once the patient’s length segment is identified, the new model follows a different pathway. The patient’s measured TBW is used to identify their HS, rather than using measured MAC (as in the original method). This is done by finding the HS (in the patient’s length segment) for which the TBW estimate is closest to the measured TBW. The LBW estimate for that HS can be read directly off the tape. Figure 2 illustrates how the new PAWPER XL-MAC tape model would function. LBW is read directly off the tape, using length measured by the tape and actual TBW as the input data, without the need for calculations.

Generation of data

The new model was used to generate estimates of weight from height and MAC for each individual in the dataset. Estimates of LBW were created in a spreadsheet (Microsoft Excel for Mac 2021) using measured TBW and height from the NHANES datasets as input data. The formulas used in the spreadsheet to calculate the estimates were custom developed. Height was used to determining the appropriate length segment of the tape to be used in the next step of the estimation process. In this second step, the TBW estimate on the tape closest to the actual TBW was established - this identified the participant’s HS category. The LBW assigned to that HS category on the tape was then used as the LBW estimate for the participant. This process was repeated to generate LBW estimates for each of the participants in the dataset.

Analysis of data and outcomes

In the data analysis, the performance of the virtual PAWPER XL-MAC estimation of LBW was compared against the participants’ actual FFM (as measured using Dual Energy X-ray Absorptiometry (DXA), the gold standard for FFM measurement). In general, FFM and LBW are considered to be equivalent, although FFM may be slightly lower than LBW, as LBW includes the lipid mass in cell membranes, the central nervous system and bone marrow [5,19]. In this study, the term FFM is used to represent DXA-measured FFM, and the term LBW is used more generally, including for estimated values.

The principal indicators of the performance were: mean percentage error (MPE) which is a measure of bias; the root mean squared percentage error (RMSPE) which is a measure of precision; and the percentage of weight estimations falling within 10% and 20% of actual measured FFM (P10 and P20 respectively) which is a measure of overall accuracy. The percentage of weight estimations falling within 10% and 20% is a standard descriptor used in weight estimation studies. It is clinically relevant as a substantial number of patients receiving a weight estimation with >10% error is at risk of harm from a medication error [10].

To be able to compare the current model with other, older LBW estimation methods, the root means square error (RMSE) was calculated. This is not a good statistic to evaluate predictive performance or compare data from different studies, as its value is dependent on the actual weight of the study participants, and the range of BMIs in the study sample. If the weights of participants in other studies differ substantially, then the value of this metric is of limited value. However, it is the only potentially useful outcome measure that has been reported in the few previously published studies.

Acceptable outcome criteria

The primary outcome measure was the performance of the model when compared to the measured FFM. A P20 >95% and a P10 >70% were considered to be acceptable accuracy of estimation, as has previously been suggested for TBW estimations [20]. This benchmark is regularly achieved in children by the paediatric dual length-based, habitus-modified weight-estimation systems.

## Results

A total of 33,215 individuals were included in the study, with 16,358 females (49.2%) and 16,857 males (50.8%). The basic demographic and anthropometric characteristics of the study population are shown in Table 1.

**Table 1:** Demographic characteristics of the participants in the study dataset. UQ = upper quartile, LQ = lower quartile, BMI = body mass index. The NHANES datasets are not fully representative of the US population, as some subgroups of age and race or ethnicity are oversampled. This dataset, therefore, contains a higher proportion of Non-Hispanic Black participants, Hispanic participants and Asian participants than the general population. However, the overall age distribution and BMI distribution are very similar to that of the general US population.

	POOLED DATASET		
	All	Male	Female
N (%)	33215		
Age (years) Median (UQ, LQ)	38 (23, 53)	37 (22, 53)	40 (24, 53)
<20 years (%)	18.2	19.6	16.8
20 to 29.9 years (%)	16.9	17.4	16.4
30 to 39.9 years (%)	16.6	16.6	16.6
40 to 49.9 years (%)	17.5	16.7	18.4
50 to 59.9 years (%)	15.2	14.6	15.7
60 to 69.9 years (%)	8.2	7.9	8.5
70 to 79.9 years (%)	4.5	4.7	4.3
>=80 years (%)	2.9	2.7	3.2
BMI (kgm^-2^) Median (UQ, LQ)	26.9 (23.2, 31.5)	27 (23.7, 30.7)	27.2 (23.0, 32.6)
<18.5 kg^-2^	2.5	2.3	2.8
18.5 to 24.9 kg^-2^	34.2	33.9	34.5
25 to 29.9 kg^-2^	31.5	35.9	26.9
30 to 34.9 kg^-2^	18.1	17.8	18.4
35 to 39.9 kg^-2^	8.1	6.4	9.8
40 to 44.9 kg^-2^	3.5	2.4	4.6
45 to 49.9 kg^-2^	1.3	0.8	1.9
>=50 kg^-2^	0.8	0.5	1.1
Ethnicity (%)			
Mexican American (%)	15.7	15.6	15.8
Other Hispanic (%)	10.3	9.4	11.2
Non-Hispanic White (%)	33.1	33.5	32.8
Non-Hispanic Black (%)	22.2	21.7	22.6
Non-Hispanic Asian (%)	14.3	15.1	13.5
Other (%)	4.4	4.7	4.1
Weight (kg) Median (UQ, LQ)	76.2 (64.2, 90.4)	81.1 (70.2, 94.2)	70.1 (59.3, 84.8)
Height (cm) Median (UQ, LQ)	167.6 (160.6, 175)	174.5 (169.3, 179.6)	161.1 (156.3, 165.9)

The results of the analyses of the performance of the new PAWPER XL-MAC tape model are shown in Table 2.

**Table 2:** Results of the weight estimation performance analyses in the pooled dataset. The results for the performance outcomes are shown by subgroups of age, weight status and ethnicity. MPE = mean percentage error, P10 = percentage of weight estimations within 10% of actual weight, P20 = percentage of weight estimations within 20% of actual weight.

	POOLED DATASET (N=33215)											
	All				Male				Female			
	MPE (%)	RMSPE (%)	P10 (%)	P20 (%)	MPE (%)	RMSPE (%)	P10 (%)	P20 (%)	MPE (%)	RMSPE (%)	P10 (%)	P20 (%)
ALL	-1.1	5.5	86.4	99.7	-1.1	5.4	87.2	99.7	-1.0	5.6	85.6	99.7
AGE												
<20 years	-2.6	5.8	84.2	99.6	-2.9	6.0	83.3	99.6	-2.2	5.6	85.3	99.7
20 to 29.9 years	-2.3	5.5	86.5	99.6	-2.3	5.4	87.3	99.5	-2.3	5.6	85.6	99.8
30 to 39.9 years	-2.2	5.4	86.4	99.5	-2.1	5.1	88.6	99.2	-2.3	5.7	84.2	99.9
40 to 49.9 years	-1.6	5.2	88.2	99.5	-1.5	5.0	89.6	99.3	-1.7	5.4	86.9	99.6
50 to 59.9 years	-0.1	5.1	88.2	99.3	-0.2	4.8	89.8	98.9	0.0	5.4	86.6	99.6
60 to 69.9 years	2.0	5.4	86.7	99.7	1.8	5.2	87.5	99.8	2.2	5.5	86.0	99.6
70 to 79.9 years	3.6	5.9	82.0	99.3	4.1	6.0	80.9	99.2	3.1	5.8	83.2	99.3
≥80 years	3.9	6.2	80.8	99.0	5.8	6.8	76.1	98.0	2.3	5.7	84.8	99.8
BMI												
<18.5 kg^-2^	-0.3	5.2	89.1	99.3	-0.6	4.9	91.3	99.0	-0.1	5.4	87.2	99.6
18.5 to 24.9 kg^-2^	-1.9	5.6	85.5	99.6	-2.7	5.6	85.4	99.5	-1.1	5.5	85.6	99.6
25 to 29.9 kg^-2^	-0.9	5.4	86.4	99.6	-0.7	5.2	87.6	99.5	-1.1	5.6	84.6	99.7
30 to 34.9 kg^-2^	-0.5	5.3	87.8	99.5	0.2	5.1	88.6	99.2	-1.2	5.5	86.9	99.8
35 to 39.9 kg^-2^	-0.1	5.5	85.4	99.3	1.2	5.5	84.6	98.4	-1.0	5.5	85.9	99.9
40 to 44.9 kg^-2^	0.3	5.5	87.1	99.4	1.1	5.2	90.8	99.0	-0.1	5.7	85.1	99.6
45 to 49.9 kg^-2^	-1.0	5.4	87.1	98.4	-2.1	5.5	84.7	96.9	-0.6	5.3	88.1	99.0
≥50 kg^-2^	-2.2	7.0	78.7	97.7	-1.7	7.6	74.1	96.3	-2.3	6.7	80.8	98.3
ETHNICITY												
Mexican American	-1.9	5.2	88.5	99.4	-1.5	4.9	90.1	98.9	-2.3	5.4	87	99.8
Other Hispanic	-2	5.2	88.2	99.2	-2.3	4.9	89.9	99.0	-1.8	5.5	86.8	99.3
Non-Hispanic White	-1.3	5.3	87.6	99.1	-0.9	5.1	88.3	98.4	-1.6	5.4	86.9	99.9
Non-Hispanic Black	-4.3	6.4	79.4	99.0	-4.4	6.4	80.0	98.4	-4.1	6.3	78.9	99.5
Non-Hispanic Asian	-0.3	5.0	89.2	99.5	0	4.7	91.1	99.4	-0.6	5.3	87.2	99.7
Other	-2.7	5.6	84.6	99.3	-2.6	5.4	86.3	99.0	-2.9	6.0	82.7	99.6

**Figure 3:**
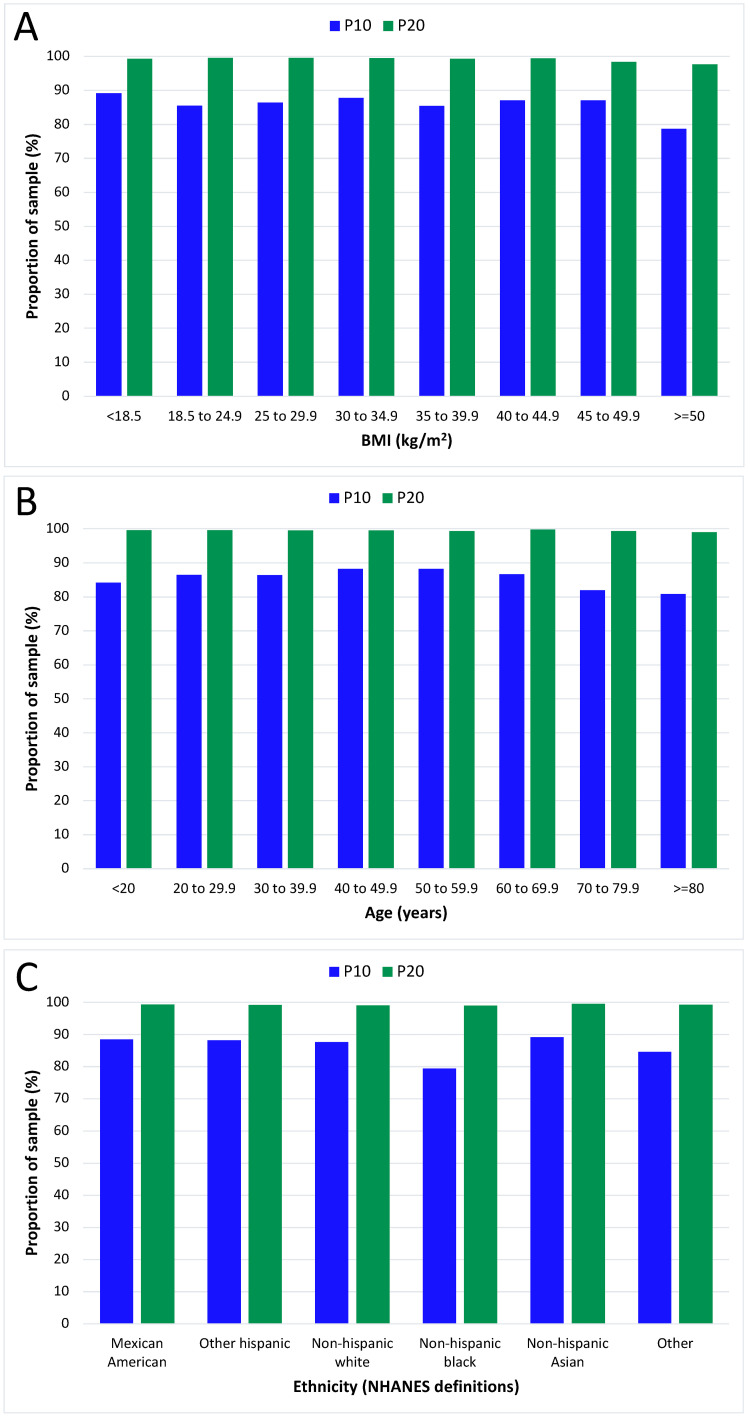
The performance of the current model, by subgroups of BMI, age, and ethnicity. The P10 and P20 outcome data (an indicator of overall performance of the model) is shown. The predetermined minimum acceptable outcome was a P10 of 70% and a P20 of 95%. This was achieved in every subgroup. Panel A shows the performance by subgroups of BMI, Panel B for subgroups of age, and Panel C for subgroups by ethnicity.

The relationship between BMI, age, and ethnicity with the overall accuracy of the model, as reflected by the P10 and P20 outcomes, is shown in Figure 3. The consistent accuracy of the model across all subgroups is notable. The model achieved the predetermined accuracy target (P20>95%, P10>70%) in every subgroup, including morbidly obese participants. The RMSE from the current study sample was 2.9 kg (95% confidence intervals 2.8, 3.0 kg).

## Discussion

It is not yet clear how we should estimate alternate weight scalars, such as LBW, for obese patients in need of urgent weight-based drug administration [21]. In clinical practice, since it is impractical to measure LBW, it must be estimated and it is, therefore, important to identify the best methods for this estimation process. Remarkably, the accuracy of methods of estimation of LBW for use in a clinical setting, particularly in relation to drug dosing, has not been well studied. In the few studies that have evaluated the accuracy of methods of estimating LBW, the methodology has been poor, with the limited presentation of data that can be compared with other studies. Furthermore, the estimation of LBW has never been studied for emergency medicine applications, where simplicity and ease of use are essential components of the estimation method. This topic is important because the inappropriate use of TBW for all weight-based drug dose calculations may cause supratherapeutic levels of hydrophilic drugs, increasing the risk of adverse events [4,22]. LBW is the best scalar to use in these patients, from a pharmacological perspective. Although IBW has been used as a dose scalar, it is not ideal especially since IBW is substantially lower than LBW in morbidly obese patients [4,19,21,23]. In addition, the biological validity of IBW is unclear, and its use should be limited when it comes to drug dose calculations.

In this study, the PAWPER XL-MAC model achieved a high level of accuracy. It remained accurate even in morbidly obese and super-obese participants. In this respect, it outperformed the original PAWPER XL-MAC methodology for LBW estimation [24]. The current model achieved a P10 of 86.4% and a P20 of 99.7%, compared to the original model with a P10 of 78.3% and a P20 of 98.3%. It was in the subgroups of morbidly obese and super-obese patients that the current model was substantially better, however, the original model had lower accuracy in these participants (especially females). It is likely that the lower accuracy of TBW estimation in morbidly obese and super-obese individuals caused the lower accuracy of LBW estimations in the original model [8]. The use of actual, measured TBW is, therefore, advisable to ensure accurate estimations of LBW, whenever possible. This is a useful finding, as morbidly obese patients are often at the highest risk of medication errors.

The original PAWPER XL-MAC methodology achieved an accuracy comparable to the current gold standard for clinical LBW estimation, the Janmahasatian formula [19,24]. The modified PAWPER XL-MAC methodology studied here exceeded the gold-standard performance. However, comparisons to the Janmahasatian formula were limited by the incomplete data reporting in studies evaluating this method. In its original validation study, the Janmahasatian formula achieved an RMSE of 4.4 kg (95% confidence intervals 3.4, 5.2 kg), and an RMSE of 5.2 kg (95% confidence intervals 4.9, 5.4 kg) in the only subsequent study to evaluate its performance [19,23]. In the current study, the PAWPER XL-MAC model achieved an RMSE of 2.9 kg (95% confidence intervals 2.8, 3.0 kg). Although this indicates a better performance than the Janmahasatian formula, these methods need to be directly assessed in future studies in a single sample to allow for a more meaningful comparison.

As a potential method to be used in emergency and critical care, a weight estimation system needs to be easy to use and must not require calculations in the methodology [25,26]. The model described in this paper is modified from a paediatric system that has been evaluated during emergencies and found to be resilient to the demands and exigencies of these situations [13,27-29]. Although future studies will need to confirm its suitability, it is likely that it will perform well during emergency care, despite the small differences in methodology.

The ability to accurately, and easily, estimate LBW has important implications for clinical care as well as for research. The use of the correct scalars has the potential to reduce medication errors in obese patients, with a reduction in patient harm [4]. In terms of research, the appropriate use of TBW or LBW could facilitate the approval of new drugs by ensuring drug safety across a wide range of patient subgroups [21]. Some authors have even posited that LBW should be used in normal-weight individuals as well and that new dosing guidelines can be developed that apply to all patients [21]. The PAWPER XL-MAC method could provide one solution to this problem by increasing access to easy, accurate, point-of-care estimates of LBW.

Limitations

This was a virtual study, in which information from a database was used as the input data. The benefit and validity of virtual studies have previously been documented [30]. Nonetheless, the use of the model in a prospective study will be needed to confirm and validate the findings of this preliminary study. Measurements of TBW may themselves not be accurate if obtained during acute or critical care, which could impact on the accuracy of this method. If a measured weight cannot be obtained, patient or family estimates could be used, but they may also be inaccurate. These are factors that also need to be evaluated in future prospective studies. Finally, not enough is known about dose scaling in obese patients and there are few drugs that have manufacturer recommendations for dose modifications in obese patients. However, having the means to easily estimate LBW in clinical practice may facilitate future changes in research and clinical protocols.

## Conclusions

This preliminary study on the use of the PAWPER XL-MAC tape method to predict LBW (when TBW is known) showed promising results. The accuracy achieved by the model exceeded the predetermined benchmark (P20>95%), with nearly 100% of estimations within 20% of measured FFM. The model was accurate in all subpopulations, including in morbidly obese and super-obese individuals. The new model outperformed the original PAWPER XL-MAC model and was at least as accurate, if not more accurate, than the Janmahasatian method, arguably the current gold standard for LBW estimation. The ability to easily estimate LBW at the patient’s bedside has the potential to increase the use of correct drug dose scaling in obese patients requiring emergency or critical care. Future prospective studies will be required to further validate this system, including during real and simulated clinical scenarios.
